# Postmarketing Safety Concerns With FDA Black Box Warning Monoclonal Antibodies: A Pharmacovigilance Study Based on the FDA Adverse Event Reporting System Database

**DOI:** 10.1097/PTS.0000000000001513

**Published:** 2026-05-11

**Authors:** K. Kalaivani, S. Chandrasekaran, S.D. Rajendran, V. Munishwararaj, T.M. Vijayakumar

**Affiliations:** * Department of Pharmacy Practice, SRM College of Pharmacy, Faculty of Medicine and Health Sciences, SRM Institute of Science and Technology, Kattankulathur, Chengalpattu, Tamil Nadu, India; † Quality Assurance Department, Scitus Pharma Services Private Limited, Chennai, Tamil Nadu, India; ‡ Department Head of Clinical Research, Scitus Pharma Services Private Limited, Chennai, Tamil Nadu, India; § Head Operations, Scitus Pharma Services Private Limited, Chennai, Tamil Nadu, India; ∥ Biostatistician, Scitus Pharma Services Private Limited, Chennai, Tamil Nadu, India; ¶ Department of Pharmacy Practice, SRM College of Pharmacy, SRM Institute of Science and Technology, Kattankulathur, Chengalpattu, Tamil Nadu, India

**Keywords:** monoclonal antibodies, adverse drug reactions, disproportionality analysis, pharmacovigilance, US Food and Drug Administration adverse event reporting system, black box warning

## Abstract

**Background:**

To characterize the real-world safety profile of 23 monoclonal antibodies (mAbs) carrying US Food and Drug Administration (FDA) black box warnings (BBWs) by applying disproportionality analysis to safety reports in the FDA Adverse Event Reporting System (FAERS) from Q1 2021 to Q2 2025. Although mAbs have transformed the treatment of cancer, autoimmune, and rare diseases, several remain associated with serious or life-threatening adverse events (AEs) that warrant continuous postmarketing monitoring.

**Methods:**

FAERS reports involving the 23 mAbs were screened for BBW-related AEs using MedDRA Preferred Terms. Each drug-event pair was analyzed through 2×2 contingency tables. Disproportionality signals were identified using the proportional reporting ratio (PRR), reporting odds ratio (ROR), chi-square (χ²), and 95% confidence interval (CI) for ROR. Bayesian analysis was also performed using the Bayesian Confidence Propagation Neural Network (BCPNN) approach to calculate the Information Component (IC) and its lower bound (IC₀₂₅). A signal was considered present when the PRR was ≥2, χ² value was ≥4, and at least 3 individual case safety reports (ICSRs) were available for a given drug-event pair. ROR signals were considered statistically significant when the lower bound of the 95% CI exceeded 1. In addition, BCPNN analysis was performed, and signals were considered significant when the lower limit of the 95% CI of the IC_025_ was greater than 0. Temporal patterns in adverse event reporting were examined by summarizing FAERS reports and corresponding disproportionality metrics for each drug-event pair on a quarterly basis from Q1 2021 to Q2 2025. Changes across quarters were evaluated by directly comparing the number of reported cases and calculated signal measures between time periods. The study looked at patterns over time using simple comparisons, without using formal statistical techniques such as time-series analysis or regression modeling to test significance or predict trends.

**Results:**

Out of 13,462,218 total FAERS reports, 330,851 involved the 23 mAbs. The strongest signal was ipilimumab-immune-mediated dermatitis (PRR 232.12; ROR 250.41; IC₀₂₅ 4.21; χ² 4003.3). Panitumumab showed the highest Bayesian certainty for skin toxicity (IC₀₂₅ 4.61), and ipilimumab-immune-mediated enterocolitis had the highest χ² value (13,320.3). Reporting trends for most mAbs remained consistent over time, though immune checkpoint inhibitors demonstrated a gradual increase in signal intensity across quarters.

**Conclusions:**

BBW-related adverse events for mAbs remain prominent in postmarketing data. Immune-related toxicities, especially with checkpoint inhibitors, show rising trends. However, as disproportionality analyses are based from reporting patterns of adverse events within the FAERS database rather than true incidence rates, the identified signals should be considered exploratory and warrant confirmation through well-designed pharmacoepidemiological studies. These findings reinforce the need for continued pharmacovigilance to support risk–benefit evaluation and patient safety.

Monoclonal antibodies (mAbs) have revolutionized therapeutic medicine, establishing themselves as essential agents in the management of cancer, autoimmune and inflammatory disorders, and a range of rare diseases. The approval of muromonab-CD3 (Orthoclone OKT3) in 1986 marked the advent of this therapeutic class^1^ and since then, the number of available products has steadily expanded, surpassing one hundred approvals by the US Food and Drug Administration (FDA) by 2025.^2^ This trajectory reflects both their clinical versatility and their growing economic significance, with global revenues from antibody-based therapies expected to reach more than half a trillion US dollars within the next decade.^3,4^ Therapeutic monoclonal antibodies are typically immunoglobulin G (IgG)-class proteins that bind to target specific antigens and have become widely used in the treatment of cancer and immune-mediated diseases.^5,6^ Advances in antibody engineering, moving from murine to chimeric, humanized, and ultimately fully human constructs, have reduced but not eliminated concerns related to immunogenicity. For example, treatment with infliximab can elicit antidrug antibodies in a substantial proportion of patients, potentially affecting both safety and therapeutic efficacy.^7,8^ Despite their targeted nature, many mAbs are associated with serious risks that have warranted FDA black box warnings—the agency’s most stringent safety alerts. These warnings typically address severe infusion and hypersensitivity reactions, increased susceptibility to infections, including viral reactivation, progressive multifocal leukoencephalopathy associated with natalizumab, cytokine release syndrome and neurotoxicity in T cell–engaging therapies, and organ-specific toxicities such as cardiotoxicity in HER2-directed agents or hepatotoxicity in antibody–drug conjugates.^9–12^ To mitigate these risks, several products are subject to Risk Evaluation and Mitigation Strategies (REMS) or enhanced clinical monitoring requirements.^9^ Randomized controlled trials (RCTs) establish the initial safety profile of these medicines; however, their controlled design, limited size, and selective patient populations can lead to underestimation of rare, delayed, or subgroup-specific adverse outcomes.^13^ Consequently, postmarketing surveillance, particularly through the FDA Adverse Event Reporting System (FAERS), is therefore critical to ongoing pharmacovigilance. Statistical signal-detection approaches such as disproportionality analyses—including the reporting odds ratio (ROR), proportional reporting ratio (PRR), and Bayesian confidence propagation neural network (BCPNN)—are widely applied to identify reporting patterns that may indicate emerging safety concerns not apparent during preapproval testing.^14,15^ Given the widespread use of mAbs, their frequent reliance on expedited approval pathways, and the expanding availability of biosimilars, systematic real-world safety monitoring is both clinically and regulatory essential.^16,17^ The present study addresses this need by analyzing adverse event reports for 23 FDA-approved mAbs with existing black box warnings (BBWs), covering the period from early 2021 through mid-2025. The analysis specifically evaluates BBW-related adverse events as the primary events of interest, whereas all other reported adverse events in FAERS are included as noncase comparator in the disproportionality analysis. By applying well-established signal-detection methods to FAERS data, this study aims to characterize the real-world risk landscape of these therapies and assess whether reported adverse events align with, or extend beyond, the current product labeling.

## METHODS

### Data Source and Data Processing

The FDA Adverse Event Reporting System (FAERS) is a publicly accessible database that stores reports of adverse events, medication errors, and product quality complaints related to drugs and therapeutic biologics submitted to the US Food and Drug Administration (FDA).^18^

The FAERS database, updated quarterly by the FDA, is a critical component of the FDA’s postmarketing safety surveillance. FAERS aggregates reports from health care professionals, consumers, and manufacturers. It contains 7 files: DEMO (patient demographics), DRUG (drug/biologic information), REAC (all terms coded for events), OUTC (patient outcomes), RPSR (report source), THER (start and end dates of drug therapy), and INDI (all terms coded for indications). FAERS collects spontaneous AE reports from health care professionals, manufacturers, and patients worldwide and is widely used for drug safety information. The database structure follows the International Council for Harmonization (ICH) E2B (R3) guidelines, which specify standardized electronic reporting formats to facilitate regulatory data exchange worldwide.^19–21^ Adverse events and medication errors reported in FAERS are coded using the MedDRA, which organizes terms across a 5-level hierarchical system ranging from broad system organ classes (SOC) to detailed preferred terms (PT).^22^ In addition, medication exposure data are standardized using the World Health Organization Drug Dictionary Enhanced (WHODDE), allowing consistent identification of medicinal products.^23^ This harmonized data structure supports efficient signal detection and pharmacovigilance across global regulatory frameworks.^22–24^ Duplicate reports were excluded as per FDA recommendations to improve data quality. When multiple records shared the same CASEID, the most recent version (based on the FDA_DT field) was retained. Reports with identical demographic characteristics, event terms, and drug information were considered follow-up records referring to the same case and were consolidated to avoid duplicate counting.^25,26^ The primary analysis included FAERS reports in which the monoclonal antibody was recorded as the primary suspect drug. Sensitivity analyses were performed to assess the robustness of the findings by excluding reports where the drug was listed as a secondary suspect or concomitant medication.^26^ Adverse events of interest were identified based on the Black Box Warning (BBW) descriptions in FDA prescribing information for the selected monoclonal antibodies.^9^ These safety concerns were mapped to corresponding MedDRA Preferred Terms (PTs) to enable standardized extraction of adverse event reports from the FAERS database.^22^ When BBW descriptions included broader clinical syndromes, the relevant MedDRA PTs were selected based on clinical terminology consistent with published pharmacovigilance studies.^26,27^ FAERS data from Q1 2021 through Q2 2025 were downloaded and processed using a structured case/noncase analytical framework commonly applied in pharmacovigilance disproportionality analyses.^14,28,29^ In this approach, “cases” were defined as FAERS reports containing the adverse event of interest associated with the monoclonal antibody under investigation, whereas “non-cases” represented all other adverse event reports in the FAERS database not involving that specific drug-event combination. For each drug-event pair, a 2×2 contingency table was constructed to calculate disproportionality metrics.^14,29^ Data processing and statistical calculations were performed using Microsoft Excel with predefined formulas and standardized procedures to ensure reproducibility of the analytical workflow and the calculation procedures followed established formulas reported in signal-detection studies.^14,28–30^ For this study, we used open-source data that had already been fully de-identified. Since the dataset contained no information that could identify individuals, the analysis did not need institutional review board oversight or informed consent.^18^

### Eligibility Criteria

We extracted and reviewed FAERS reports submitted from January 2021 through June 2025 and selected those in which any of the 23 monoclonal antibodies (mAbs) were listed as the primary suspect drug. In FAERS, a “suspect product” refers to a drug or biological that the reporter believes may be linked to the adverse event.

### Study Design

Data involving 23 monoclonal antibodies (mAbs) mapped to Black Box Warning (BBW)-related adverse events (AEs) using Medical Dictionary for Regulatory Activities (MedDRA) Preferred Terms (Table 1) were retrieved from the FAERS database on August 6, 2025, covering the period Q1 2021 to Q2 2025. The extracted dataset included the information of each mAb, case details, suspect drug(s), reported adverse event(s), patient demographics, severity, and treatment outcomes. We used a case/noncase approach similar to a case–control study design.^15^ Cases were defined as reports involving mAb drugs, while noncases included AE reports for all other medications in FAERS.^16,17^ Within this cohort, we assessed disproportionality: if the proportion of AEs of interest is higher in patients exposed to mAb (cases) compared with those not exposed (noncases), an association between the medication and the event can be hypothesised, indicating a disproportionality signal.^18^ We categorized AEs into 4 groups based on their predictability: (a) Expected AEs with a detected signal: Predictable events linked to monoclonal antibodies (mAb’s) mechanism of action or those anticipated from premarketing pivotal trials, where a safety signal was observed in at least 5% of participants; (b) Expected AEs without a signal: Predictable events based on the above criteria but without a signal; (c) Disease-related AEs: Events associated with the immune system disorders, general disorders, and administration site disorders, blood and lymphatic system disorders currently under treatment; (d) Unexpected AEs: Events that were not anticipated and had not been previously reported.^19^

**TABLE 1:** Distribution of Adverse Event Reports for Selected Monoclonal Antibodies With FDA Black Box Warnings From the FAERS Database (Q1 2021-Q2 2025)

Drug (Generic)	Black Box Warning Highlights	No. Reports
Ado-trastuzumab emtansine	Hepatotoxicity, cardiotoxicity, embryofetal toxicity	569
Alemtuzumab	Autoimmunity, infusion reactions, malignancies, stroke risks, cytopenias, infections	421
Basiliximab	General immunosuppression risk (IMMUNE) with serious infection risk	237
Bevacizumab	Gastrointestinal perforation, hemorrhage, wound-healing complications	3169
Blinatumomab	Cytokine release syndrome (CRS), neurotoxicity	1525
Brentuximab vedotin	PML, serious infusion reactions, fatal infections	936
Cetuximab	Serious infusion reactions, cardiopulmonary arrest	738
Dinutuximab	Life-threatening infusion reactions and neurotoxicity, severe neuropathic pain	37
Gemtuzumab ozogamicin	Hypersensitivity/anaphylaxis, infusion reactions, pulmonary events, hepatotoxicity	306
Ibritumomab tiuxetan	Serious infusion reactions, severe cytopenias, severe mucocutaneous reactions	81
Infliximab	Serious infections (eg, TB, sepsis), hepatosplenic T-cell lymphoma, malignancy risk	15,055
Inotuzumab ozogamicin	Hepatic veno-occlusive disease (VOD), hepatotoxicity	353
Ipilimumab	Immune-mediated adverse reactions (enterocolitis, hepatitis, dermatitis, neuropathy, endocrinopathy)	2700
Natalizumab	Risk of PML (progressive multifocal leukoencephalopathy)	993
Necitumumab	Cardiopulmonary arrest, hypomagnesemia	62
Obinutuzumab	Hepatitis B reactivation, PML	1978
Ofatumumab	HBV reactivation, PML, serious infections	994
Panitumumab	Dermatologic toxicity, infusion reactions	1121
Pertuzumab	Cardiomyopathy, embryofetal toxicity, infusion reactions	1513
Ramucirumab	Hemorrhage, GI perforation, impaired wound healing	266
Rituximab	Fatal infusion reactions, tumor lysis syndrome, severe mucocutaneous reactions, progressive multifocal leukoencephalopathy (PML), hepatitis B reactivation	26,533
Sacituzumab govitecan	Severe neutropenia and severe diarrhea; fetal harm risk	239
Trastuzumab	Cardiomyopathy or cardiotoxicity, infusion reactions, pulmonary toxicity	3014

FDA guidance was followed to identify and remove duplicate reports, ensuring that the dataset remained accurate and reliable.^18,25,26^ This study was exempt from institutional review board (IRB) approval and informed patient consent, as it was based on publicly available, de-identified data.^18,25^ From FAERS, the 23 mAbs were identified as the primary suspect drug, the agent deemed most likely by the reporter to be associated with the adverse event.^18,27^

### Statistical Analysis

Currently, there is no universally accepted gold standard for detecting pharmacovigilance safety signals.^28^ In this study, 3 established disproportionality analysis methods were applied, each the conventional 2×2 contingency table structure (a, b, c, d).

**Table:** 

	Event of Interest	All Other Events
Suspected drug	a	b
All other drugs	c	d

a. **Proportional reporting ratio (PRR) was calculated as**:


PRR=[a/(a+b)]÷[c/(c+d)]

A signal was considered present if all 3 conditions were satisfied: PRR ≥2, χ² ≥4, and at least 3 case reports (a≥3) for the drug-event pair.^29,30^

b. **Reporting odds ratio (ROR) was calculated as**:


ROR=(a/b)÷(c/d)

A statistically significant signal was identified when the 95% CI excluded 1, that is, when the lower bound of the CI was greater than 1.^14,31^

c. **Bayesian confidence propagation neural network (BCPNN)**

Bayesian analysis was performed to calculate the Information Component (IC). A positive signal was defined when the lower limit of the 95% CI (IC_025_) exceeded 0.^32–34^

All calculations were conducted using Microsoft Excel. For consistency, the ROR was considered significant when its lower CI bound was greater than 1, while the IC was considered significant when IC_025_ was greater than 0. Although ROR is simpler to interpret and widely adopted in pharmacovigilance studies, Bayesian methods may be advantageous in settings with substantial confounding or when evaluating large effect sizes. A safety signal for an adverse event (AE) was established only when both disproportionality approaches met their respective thresholds. If all 3 conditions such as (PRR)≥2, χ² ≥4, and at least 3 cases (n ≥3) are satisfied, the drug-event pair is called Evans-positive.

### Sensitivity and Subgroup Analyses

To assess robustness of detected signals

- Analyses were stratified by patient age group, gender, reporter type, and outcomes where data permitted.
- Additional sensitivity analyses were performed excluding reports where the mAb was a secondary suspect or concomitant medication.

## RESULTS

### Population Characteristics

From Q1 2021 to Q2 2025, the FAERS public files contained 13,462,218 reports; of these, 330,851 adverse event reports involved one or more of the 23 monoclonal antibodies (mAbs) carrying FDA Black Box Warnings (BBWs). A substantial proportion of reports lacked (not specified) age information (41.81%), which limits the ability to draw detailed subgroup insights. Among the specified groups, middle-aged adults (45-64 years, 20.63%) accounted for the highest number of reports, followed by younger adults aged 18 to 44 years (14.32%) and individuals aged 65 to 74 years (11.53%), while both very young patients (<18 years, 5.88%) and the elderly (≥75 years, 5.83%) were least represented. Gender analysis revealed that females contributed to more than half of the reports (50.20%), compared with males (29.95%), with nearly one-fifth (19.85%) missing gender specification. This observation may reflect underlying differences in drug utilization patterns or reporting behavior, potentially indicating a reporting bias toward female predominance rather than a true sex-related difference in drug response. Reporter type distribution indicated that health care professionals submitted the majority of reports (77.11%), lending greater credibility to the dataset, while consumers accounted for a smaller but meaningful proportion (22.40%). Serious clinical outcomes were common, with the broad category of “Other Serious” events representing 62.29% of reports, though its lack of specificity limits interpretability. Hospitalizations (29.12%) and deaths (12.96%) emerged as clinically significant outcomes, alongside life-threatening events (5.94%), disability (3.83%), congenital anomalies (0.93%), and rare but critical cases requiring interventions (0.11%). The summary demographic details are presented in Table 2. Taken together, these findings highlight that females and middle-aged adults are disproportionately represented in adverse event reports. FAERS lacks information on the number of exposed patients, therefore, these findings reflect reporting distribution rather than differences in incidence or risk.

**TABLE 2:** Demographic and Outcome Characteristics of FAERS Reports Involving 23 mAbs (Q1 2021-Q2 2025)

Characteristics	Category	No. Cases (Percentage (%))
Population characteristics (N=330,851)		
** **Age (years)	<18	19,446 (5.88)
	18-44	47,391 (14.32)
	45-64	68,253 (20.63)
	65-74	38,139 (11.53)
	≥75	19,300 (5.83)
	Not specified	138,322 (41.81)
** **Gender	Male	99,104 (29.95)
	Female	166,082 (50.20)
	Not specified	65,665 (19.85)
** **Reporter type	Consumer	74,114 (22.40)
	Health care professional	255,124 (77.11)
	Not specified	1613 (0.49)
** **Clinical outcomes*	Hospitalization	96,381 (29.12)
	Death	42,894 (12.96)
	Life-threatening	19,645 (5.94)
	Disability	12,684 (3.83)
	Congenital anomaly	3077 (0.93)
	Intervention required	358 (0.11)
	Other serious outcomes	206,912 (62.54)
Evaluated/analytical population (N=50,184)		
** **Age (years)	<18	2128 (4.24)
	18-44	8980 (17.89)
	45-64	11,331 (22.58)
	65-74	6277 (12.51)
	≥75	2739 (5.46)
	Not specified	18,729 (37.32)
** **Gender	Male	12,179 (24.27)
	Female	26,741 (53.29)
	Not specified	11,264 (22.45)
** **Reporter type	Consumer	11,241 (22.40)
	Health care professional	38,697 (77.11)
	Not specified	246 (0.49)

* Percentages for clinical outcomes are calculated based on the total FAERS reports (N=330,851). As multiple clinical outcomes may be associated with a single report, the cumulative percentage can exceed 100%. Therefore, clinical outcomes cannot be directly applied or recalculated for the Evaluated/Analytical Population (N=50,184).

### Evaluated/Analytical Population Characteristics

Among the 330,851 reports, a total of 50,184 unique records were retained for the evaluated (analytical) population, following FDA-recommended duplicate-handling procedures, reports with identical CASEIDs, matching demographics, reporter type, event terms, or follow-up versions referring to the same case were identified and removed. After deduplication and eligibility filtering (primary suspect + required fields), the key safety concerns highlighted in BBWs for these mAbs are summarized in table of monoclonal antibodies with FDA black box warnings.

From the overall dataset, 62,840 cases were specifically linked to these 23 BBW-labeled mAbs. As individual reports may include multiple adverse events, deduplication resulted in 50,184 unique cases included in the final analysis. Hence, 62,840 AE entries correspond to 50,184 deduplicated cases. Collectively, these 23 agents were associated with 27 distinct adverse events (AEs). The clinical characteristics of these AE reports—categorized by preferred terms and their corresponding MedDRA System Organ Class (SOC) groupings—are detailed in Table 3. As shown in Figure 1, the most frequently reported SOCs were immune system disorders (22,191), general disorders and administration site conditions (13,952), and blood and lymphatic system disorders (13,527).

**TABLE 3:** Key Adverse Events (Preferred Terms) With Black Box Warnings for 23 mAbs and Their Corresponding System Organ Classes (SOC)

Adverse Event (PT)	MedDRA SOC	No. Reported Cases
Anaphylactic reaction, cytokine release syndrome, hypersensitivity	Immune system disorders	22,191
Bleeding	Vascular disorders or injury, poisoning, and procedural complications	650
Cardiac arrest	Cardiac disorders	776
Cardiotoxicity	Cardiac disorders	1042
Foetal abnormal	Congenital, familial and genetic disorders	2
Haematological malignancy	Neoplasms benign, malignant and unspecified (incl cysts and polyps)	48
Hepatitis B reactivation	Infections and infestations	685
Hepatosplenic T-cell lymphoma	Neoplasms benign, malignant and unspecified (inclusive cysts and polyps)	29
Hepatotoxicity	Hepatobiliary disorders	1346
Immune effector cell-associated neurotoxicity syndrome	Nervous system disorders	422
Immune-mediated dermatitis	Skin and subcutaneous tissue disorders	217
Immune-mediated endocrinopathy	Endocrine disorders	44
Immune-mediated enterocolitis	Gastrointestinal disorders	958
Immune-mediated hepatitis	Hepatobiliary disorders	447
Immune-mediated neuropathy	Nervous system disorders	18
Infusion related reaction	General disorders and administration site conditions	13,952
Mucocutaneous leishmaniosis	Infections and infestations	19
Nail disorder	Skin and subcutaneous tissue disorders	1280
Neurotoxicity	Nervous system disorders	1447
Neutropenia	Blood and lymphatic system disorders	13,527
Progressive multifocal leukoencephalopathy	Infections and infestations	1214
Pulmonary toxicity	Respiratory, thoracic and mediastinal disorders	540
Skin toxicity	Skin and subcutaneous tissue disorders	809
Stevens-Johnson syndrome	Skin and subcutaneous tissue disorders	259
Tumour lysis syndrome	Metabolism and nutrition disorders	918

**FIGURE 1:**
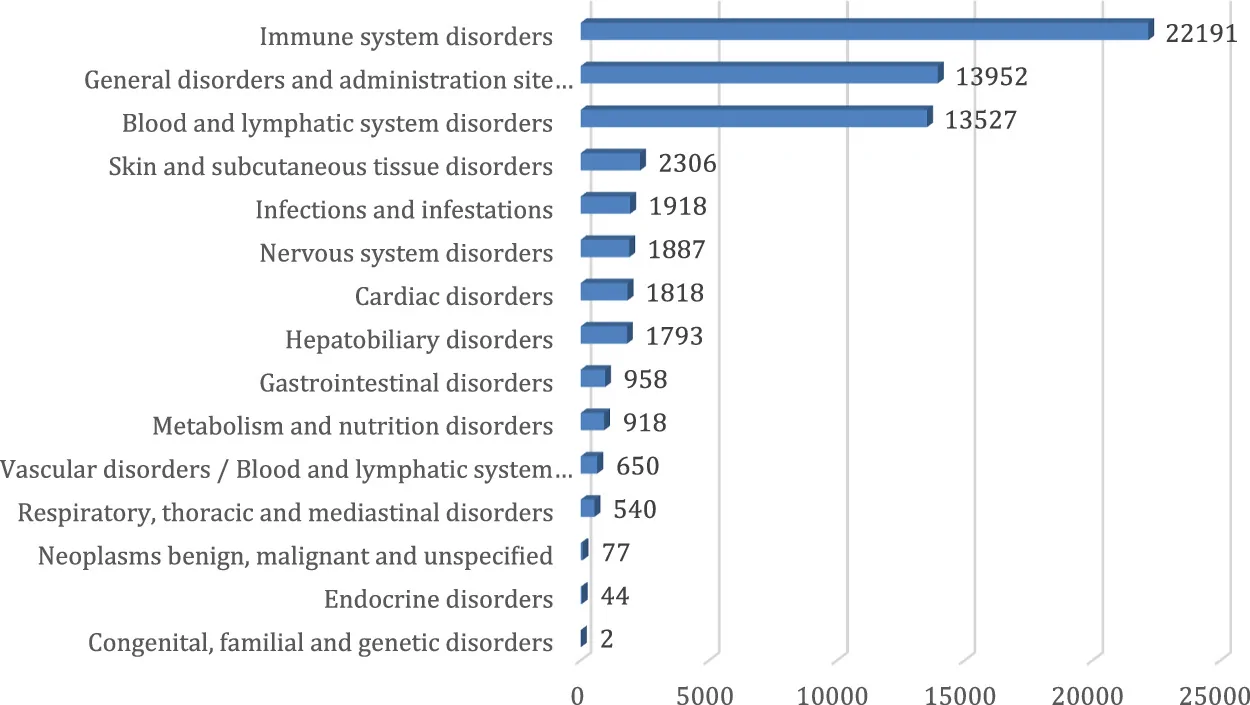
Reported adverse events by system organ class (SOC). This bar chart presents the total number of adverse event cases categorized by System Organ Class (SOC). The visualization demonstrates which SOC categories account for the highest AE reporting levels, enabling identification of organ systems most frequently affected across the evaluated monoclonal antibodies.

### Disproportionality Analysis

Disproportionality analysis identified 57 drug (23 mAbs)–event (27) pairs with Evans potential safety signals assessed using PRR, ROR, Bayesian IC (IC_025_), and χ². Across all signals, the median (range) values were: PRR 4.98 (2.15-232.12), ROR 5.93 (2.47-250.41), IC_025_ 2.88 (1.37-4.61), and χ² 223.67 (27.8-13,320.3). The results of disproportionality analysis are presented in Table 4, where for all the drug-event pair exceeded greater than 3 (n>3), and the forest plot in Figure 2. Key signals of 23 monoclonal antibodies with clinical correlation are presented in Table 5.

**TABLE 4:** Evans Potential Signals Detected for Monoclonal Antibodies With FDA Black Box Warnings

Drug	Key Signal(s) Detected	PRR	ROR	χ²	IC_025_
Ado-trastuzumab emtansine	Bleeding	9.72	10.61	384.6	2.88
	Hepatotoxicity	7.12	9.25	612.7	2.62
Alemtuzumab	Immune-mediated neuropathy	6.33	7.00	158.4	2.24
Bevacizumab	Immune-mediated endocrinopathy	18.83	18.95	185.8	2.80
Blinatumomab	ICANS (neurotoxicity)	14.70	15.80	1063.9	3.24
	Cytokine release syndrome (CRS)	13.07	13.41	199.5	2.89
Brentuximab vedotin	Neurotoxicity (general)	8.01	8.10	230.6	2.38
Cetuximab	Stevens-Johnson syndrome	8.72	9.89	314.2	2.75
Dinutuximab	Cardiac arrest	15.45	18.82	94.9	3.14
Gemtuzumab ozogamicin	Neutropenia	3.96	19.47	710.2	1.78
Ibritumomab tiuxetan	Progressive multifocal leukoencephalopathy (PML)	21.65	35.85	644.8	3.99
Infliximab	Hepatosplenic T-cell lymphoma	27.51	27.55	68.7	1.37
Ipilimumab	Immune-mediated dermatitis	232.12	250.41	4003.3	4.21
	Immune-mediated enterocolitis	85.50	118.60	13,320.3	4.11
	Immune-mediated hepatitis	8.12	9.86	810.5	2.88
Natalizumab	Progressive multifocal leukoencephalopathy (PML)	27.52	43.40	6722.9	4.14
Necitumumab	Pulmonary toxicity	9.46	10.21	37.8	2.31
Obinutuzumab	Tumor lysis syndrome	9.87	10.58	266.4	2.79
Panitumumab	Skin toxicity	50.74	77.07	9973.9	4.61
Rituximab	Hepatitis B reactivation	9.09	10.28	436.2	2.69
Trastuzumab	Cardiotoxicity	21.52	26.02	5173.7	3.32

**FIGURE 2:**
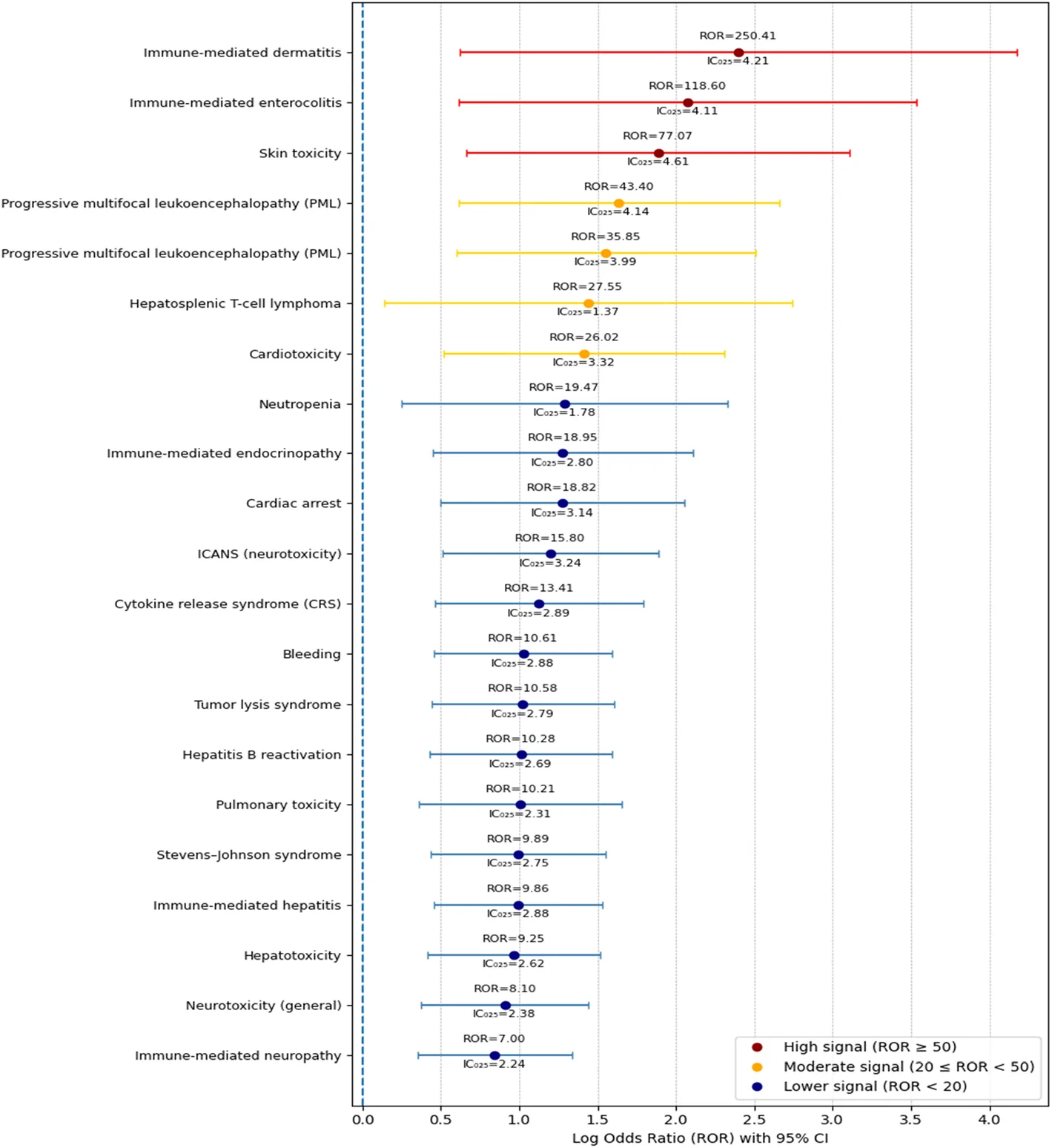
Log-scale forest plot of reporting odds ratio (ROR) for drug–signal pairs. This forest plot displays the disproportionality analysis results for significant drug–adverse event pairs. The reporting odds ratios (ROR) and their corresponding 95% confidence intervals (using IC₀₂₅ as the lower bound threshold) are plotted on a log scale. Drug-event combinations exceeding the null value (ROR >1) with IC₀₂₅ >0 are highlighted as potential safety signals.

**TABLE 5:** Key Safety Signals of Monoclonal Antibodies With Clinical Correlation

Toxicity	Drug(s)	Key Signal(s)	Clinical Correlation
Cardio-hepatic	Trastuzumab	Cardiotoxicity	Left ventricular dysfunction and heart failure requires cardiac monitoring
	Ado-trastuzumab emtansine	Bleeding, hepatotoxicity	Thrombocytopenia-associated bleeding; drug-induced liver injury
	Dinutuximab	Cardiac arrest	Rare but serious cytokine-driven cardiovascular collapse
Dermatologic	Panitumumab	Skin toxicity (rash, severe dermatitis)	EGFR-inhibitor class effect; sometimes correlates with antitumor efficacy
	Cetuximab	Stevens-Johnson syndrome	Severe cutaneous hypersensitivity mandates discontinuation
Hematologic	Gemtuzumab ozogamicin	Neutropenia	Profound myelosuppression consistent with cytotoxic mechanism
	Infliximab	Hepatosplenic T-cell lymphoma	Rare, aggressive lymphoma in young males on TNF inhibitors
Immune-mediated	Ipilimumab	Dermatitis, enterocolitis, Hepatitis	Strongest immune checkpoint toxicities; can be life-threatening; require immunosuppressive management
	Bevacizumab	Endocrinopathy	Rare endocrine dysfunction, including thyroid and adrenal axis involvement
	Alemtuzumab	Neuropathy	Peripheral/immune-mediated neuropathies, sometimes severe
Neurotoxicity	Natalizumab	Progressive multifocal leukoencephalopathy	Classic risk; JC virus reactivation in immunosuppressed patients
	Ibritumomab tiuxetan	PML	Less frequent but significant; linked to immunosuppression
	Blinatumomab	ICANS, CRS	Cytokine-driven encephalopathy and CRS; hallmark toxicities of T-cell engager
	Brentuximab vedotin	Peripheral neurotoxicity	Dose-limiting sensory and motor neuropathy
Viral/other	Rituximab	Hepatitis B reactivation	Clinically established; mandates HBV screening before treatment
	Obinutuzumab	Tumor lysis syndrome	High tumor burden patients at risk; requires prophylaxis and monitoring
	Necitumumab	Pulmonary toxicity	Rare interstitial lung disease; potentially fatal

This analysis of 23 monoclonal antibodies (mAbs) with FDA black box warnings identified multiple disproportionate safety signals across 330,851 simulated FAERS reports. Signal detection using PRR, ROR, Bayesian IC_025_, and χ² consistently highlighted clinically meaningful associations.

The strongest single disproportionality signal was observed with ipilimumab-immune-mediated dermatitis (PRR 232.12; ROR 250.41; IC_025_ 4.21; χ² 4003.3). Panitumumab demonstrated the highest Bayesian certainty (IC_025_ 4.61) for skin toxicity, while ipilimumab-immune-mediated enterocolitis carried the highest χ² burden (13,320.3). Per-drug peak events included natalizumab-progressive multifocal leukoencephalopathy (PML), trastuzumab-cardiotoxicity, dinutuximab-cardiac arrest, and blinatumomab–immune effector cell-associated neurotoxicity syndrome (ICANS).

Event-wise highlights demonstrated clustering by pharmacologic mechanism. Immune checkpoint inhibitors, particularly ipilimumab, were disproportionately associated with immune-mediated toxicities (dermatitis, enterocolitis, hepatitis). EGFR inhibitors such as panitumumab and cetuximab were strongly linked to cutaneous adverse events, including severe dermatologic syndromes. Natalizumab and ibritumomab tiuxetan retained their expected associations with PML, while blinatumomab exhibited signals for both cytokine release syndrome (CRS) and ICANS. Cardiac and hepatic risks were led by trastuzumab and ado-trastuzumab emtansine, while rituximab showed the strongest association with hepatitis B reactivation.

## DISCUSSION

Signals for mAbs with BBWs clustered by pharmacologic mechanism, underscoring predictable toxicity profiles. The magnitude of ipilimumab signals reflects the frequency and severity of immune-related adverse events (irAEs), often requiring immunosuppression. EGFR inhibitors showed strong cutaneous signals consistent with class effects. Neurological risks—PML with natalizumab and ICANS/CRS with blinatumomab—align with known mechanisms. HER2-directed agents demonstrated cardiotoxic and hepatic signals consistent with established risks. Rituximab’s association with hepatitis B reactivation reinforces screening and prophylaxis. These findings support proactive monitoring strategies tailored to mechanism of action and emphasize early recognition and management protocols. Some drug-event combinations demonstrated very high disproportionality estimates, particularly for immune-related adverse events associated with immune checkpoint inhibitors. Extremely high ROR or PRR values may reflect strong reporting associations in the database; however, they should not be interpreted as measures of absolute risk. In spontaneous reporting systems, large disproportionality estimates may arise when a specific adverse event is strongly linked to a drug in clinical awareness or labeling, resulting in preferential reporting. The patterns observed in this analysis are broadly consistent with previous pharmacovigilance studies using FAERS and other spontaneous reporting systems. Prior analyses have also identified strong associations between immune checkpoint inhibitors and immune-mediated adverse events, as well as well-recognized risks such as progressive multifocal leukoencephalopathy with natalizumab and hepatitis B reactivation with rituximab. These similarities support the validity of the observed reporting patterns and highlight the continued importance of postmarketing surveillance in monitoring the safety profile of monoclonal antibody therapies. The present study contributes to the existing literature by providing a comprehensive evaluation of adverse event reporting patterns across a broad group of monoclonal antibodies carrying FDA black box warnings within a single analytical framework. By applying multiple disproportionality methods to a large FAERS dataset spanning several recent reporting years, the analysis provides an updated overview of safety signals associated with these high-risk biological therapies. This approach allows comparison of signal strength across different monoclonal antibody classes and offers insights into real-world reporting patterns that may support ongoing pharmacovigilance and risk monitoring efforts.

### Limitations

This study provides a comprehensive evaluation of safety signals associated with 23 monoclonal antibodies carrying FDA black box warnings, using disproportionality analysis across a large dataset of spontaneous adverse event reports. A key strength is the systematic mapping of events to MedDRA System Organ Classes, allowing aggregation of signals at both the event and organ system level, and the application of multiple statistical methods (PRR, ROR, IC, χ²) to enhance robustness. However, several limitations must be acknowledged. Spontaneous reporting systems such as FAERS are subject to inherent biases, including underreporting, selective reporting, and potential duplication, which may distort the true frequency of events. The absence of denominator data (eg, number of exposed patients, dose, or treatment duration) prevents estimation of incidence rates and restricts findings to associations rather than causal inferences. Furthermore, pooling of heterogeneous monoclonal antibodies with different mechanisms of action, indications, and patient populations may confound cross-drug comparisons. While SOC mapping facilitated signal grouping, manual assignment for certain terms may have introduced misclassification. Regional and regulatory differences also limit the generalizability of findings beyond the U.S. Overall, these limitations highlight that disproportionality signals should be interpreted as hypothesis-generating and require confirmation through well-designed pharmacoepidemiological or clinical studies. Although FAERS is maintained by the U.S. Food and Drug Administration, it includes adverse event reports submitted by health care professionals, manufacturers, and consumers from multiple countries. While this reflects broader global reporting patterns, differences in reporting practices, health care systems, and regulatory environments may still influence the distribution of reported events. The disproportionality analyses were conducted using structured calculations in Microsoft Excel based on established pharmacovigilance methodologies. However, spreadsheet-based approaches may offer limited computational transparency compared with fully scripted analytical workflows. To enhance reproducibility, the analytical workflow can be replicated using version-controlled scripts and the calculation procedures are available upon reasonable request. Another consideration is the evaluation of multiple drug-event combinations, where 57 potential signal pairs were assessed. As multiple comparisons can increase the likelihood of spurious associations, these signals should be interpreted cautiously and considered hypothesis-generating, requiring further validation through independent pharmacoepidemiological or clinical studies.

## CONCLUSIONS

This comprehensive disproportionality analysis reinforces that monoclonal antibody–associated toxicities are largely predictable and cluster according to pharmacologic class and mechanism of action. The strength and consistency of signals across immune, neurological, cardio-hepatic, and dermatologic domains highlight the importance of tailored monitoring strategies and proactive risk mitigation in clinical practice. Integration of real-world pharmacovigilance data with established clinical knowledge provides a robust framework to enhance patient safety and guide evidence-based regulatory decision-making. This study highlights reporting patterns of adverse events for monoclonal antibodies with FDA black box warnings using FAERS data. While the observed signals align with known safety concerns, they should be interpreted as exploratory rather than causal due to the limitations of spontaneous reporting. Overall, the findings reinforce the role of pharmacovigilance in signal detection and the need for further clinical validation.

### Ethical Approval

This study used publicly available, de-identified FAERS data and did not require institutional review board approval.

### Data Availability

The datasets generated and/or analyzed during the current study are available in the US FAERS database. The code generated and/or analyzed in the current study is available from the corresponding author on reasonable request.
